# Atorvastatin reduces serum HMGB1 levels in patients with hyperlipidemia

**DOI:** 10.3892/etm.2012.732

**Published:** 2012-10-01

**Authors:** DAOQUN JIN, YONGBO WU, LIN ZHAO, JIE GUO, KAI ZHANG, ZHIQIANG CHEN

**Affiliations:** Department of Cardiology, Central Hospital of Huangshi, Huangshi, P.R. China

**Keywords:** high mobility group box 1 protein, inflammation, atorvastatin, hyperlipidemia

## Abstract

High mobility group box 1 protein (HMGB1) has been identified as a novel pro-inflammatory cytokine in coronary artery disease. This study investigated the effect of atorvastatin on serum HMGB1 levels in patients with hyperlipidemia. In 72 patients with hyperlipidemia, serum total cholesterol (TC), triglycerides (TG), high-density lipoprotein cholesterol (HDL-C), low-density lipoprotein cholesterol (LDL-C) and high-sensitivity C-reactive protein (hs-CRP) were compared with the levels in 32 control patients. In hyperlipidemic patients, serum HMGB1 levels were also determined by ELISA before and after a 3-month treatment of atorvastatin (20 mg/day). TC and LDL-C levels in the hyperlipidemic group (6.37±0.94 and 4.99±0.75 mmol/l, respectively) were significantly higher compared to those in the control group (4.34±0.89 and 2.57±0.82 mmol/l, respectively) (both P<0.05). Hs-CRP and HMGB1 levels in the hyperlipidemic group (3.91±1.06 mg/l and 5.42±1.56 ng/ml, respectively) were also significantly higher compared to those in the control group (1.53±0.45 mg/l and 2.11±0.95 ng/ml, respectively) (both P<0.05). After treatment with atorvasatin for three months, TC and LDL-C levels in the hyperlipidemic group were significantly decreased compared to those prior to treatment (TC, 4.67±0.89 vs. 6.37±0.94 mmol/l and LDL-C, 2.75±0.92 vs. 4.99±0.75 mmol/l, respectively) (both P<0.05). HMGB1 and hs-CRP levels in the hyperlipidemic group (3.07±1.24 ng/ml and 1.87±0.79 mg/l, respectively) were also significantly decreased compared to levels prior to treatment (5.42±1.56 ng/ml and 3.91±1.06 mg/l, respectively) (both P<0.05). Serum HMGB1 levels are increased in patients with hyperlipidemia which could be reduced by atorvastatin.

## Introduction

Atherosclerosis is characterized by a complex multifactorial pathophysiology and was previously considered as a lipid accumulation disease with an ongoing inflammatory response which was mainly attributed to hyperlipidemia. Vascular inflammation and hyperlipidemia play a predominant role in the initiation, progression and the final steps of atherosclerosis (1–3). It has been shown by previous study that hyperlipidemia promotes the release of pro-inflammatory cytokines, such as tumor necrosis factor-α (TNF-α), interleukin-6 (IL-6) and C-reactive protein (CRP) (4) indicating that hyperlipidemia promotes an inflammatory response consequently enhancing the development of atherosclerosis.

High mobility group box 1 protein (HMGB1) is a highly conserved nuclear protein that has DNA-binding properties. Several studies have revealed that HMGB1 is a necessary and sufficient mediator of lethal inflammation and functions as a novel pro-inflammatory cytokine (5–7). Recently, an elevated HMGB1 level was also detected in patients with atherosclerotic coronary artery diseases (8–10). As hyperlipidemia has been shown to promote the release of HMGB1 (11), hyperlipidemia may be involved in the inflammation process of atherosclerosis by stimulating the release of HMGB1. However, whether serum HMGB1 levels are increased in patients with hyperlipidemia remains unknown. The major aim of this study was to investigate the change in serum HMGB1 levels and the effect of atrorvastatin on HMGB1 levels in patients with hyperlipidemia.

## Materials and methods

### Study subjects

The clinical protocol was approved by the Institutional Medical Ethics Committee of the Central Hospital of Huangshi and conducted according to the ethical guidelines outlined in the Declaration of Helsinki. A total of 72 consecutive patients from the Department of Cardiology of the Central Hospital of Huangshi, China, were enrolled in this study. Patients were allocated into two groups: hyperlipidemic group [low-density lipoprotein cholesterol (LDL-C) ≥4.14 mmol/l, n=72] and control group [LDL-C <4.14 mmol/l, n=32] that was not receiving treatment with statins or other drugs for reducing lipids. All patients in this study underwent coronary angiography after admission. The patients without coronary artery stenosis served as a control group without hyperlipidemia. Exclusion criteria included patients with coronary artery disease, cardiac dysfunction, fever, peripheral vascular disease, liver or renal dysfunction, autoimmune disease, cancer, surgery or stroke within 6 months, a history of infection, chronic inflammation, abnormal thyroid function, imbalance of electrolytes and use of anti-inflammatory drugs such as corticoids, statins and other drugs for reducing lipid as well as nonsteroidal anti-inflammatory drugs excluding aspirin. The hyperlipidemic group received a treatment of 20 mg/day atorvastatin for 3 months. After three months, the serum lipids, high-sensitivity CRP (hs-CRP) and HMGB1 of the patients were measured again.

### Sample collection and biochemical investigation

Peripheral venous blood was drawn from the antecubital vein after a 12-h fasting period. Serum samples were aliquoted and stored at −70°C until used. All samples were thawed only once. Serum total cholesterol (TC), triglycerides (TG), high-density lipo-protein cholesterol (HDL-C) and LDL-C were measured using standard laboratory techniques on a Hitachi 912 Analyzer (Roche Diagnostics, Germany). Serum hs-CRP was measured by ELISA. Serum HMGB1 levels were determined with a commercially available ELISA kit (HMGB1 ELISA Kit II; Shino-Test Corporation, Tokyo, Japan) according to the kit protocol.

### Statistical analysis

Statistical analysis was performed with the SPSS 13.0 (SPSS Inc., Chicago, IL, USA). All continuous values are expressed as means ± SD. The Chi-square test or Fisher’s exact test was used to compare proportions. The Student’s t-test or paired t-test was used for comparisons between two groups. Statistical significance was defined as P<0.05.

## Results

### Clinical characteristics of the patients

There were no significant differences in age, gender, smoking status, drinking status, incidence of diabetes and hypertension and medication intake and body mass index (BMI) between the two groups. The TC and LDL-C levels in the hyperlipidemic group were significantly higher than those in the control group (both P<0.05). Hs-CRP and HMGB1 levels in the hyperlipidemic group were significantly higher than those in the control group (both P<0.05) (Table I).

### Effect of atorvastatin on hs-CRP and HMGB1 levels

Following treatment with atorvasatin for three months, TC and LDL-C levels in the hyperlipidemic group were significantly decreased compared to those prior to treatment (TC, 4.67±0.89 vs. 6.37±0.94 mmol/l and LDL-C, 2.75±0.92 vs. 4.99±0.75 mmol/l, respectively; both P<0.05). HMGB1 and hs-CRP levels in the hyperlipidemic group (3.07±1.24 ng/ml and 1.87±0.79 mg/l, respectively) were also significantly decreased compared to levels prior to treatment (5.42±1.56 ng/ml and 3.91±1.06 mg/l, respectively; both P<0.05).

## Discussion

In 1999, Wang *et al* (5) initially reported that HMGB1 functions as a pro-inflammatory cytokine in sepsis. More recently, HMGB1 was also identified as a novel pro-inflammatory mediator in atherosclerotic coronary artery diseases (8–10). A previous study demonstrated that HMGB1 is expressed abundantly in atherosclerotic plaques derived from human autopsy specimens (12). Recently, Inoue *et al* (13) showed that activated vascular smooth muscle cells are the source of HMGB1 in human advanced atherosclerotic lesions and that HMGB1 directly facilitates the production of CRP which is an independent predictor of the extent of coronary artery stenosis for patients with atherosclerotic coronary artery diseases (14,15). Meanwhile, Hu *et al* (8) showed that HMGB1 is related to the severity of coronary artery stenosis. These findings suggest that the release of HMGB1 plays an important role in promoting inflammation and the genesis of atherosclerosis. In the present study, we found that HMGB1 was increased in patients with hyperlipidemia. Haraba *et al* (11) showed that hyperlipidemia stimulates the extracellular release of nuclear HMGB1 while reducing hyperlipidemia decreases the expression of HMGB1. This indicates that increased serum HMGB1 levels may be attributed to hyperlipidemia.

In addition, we found that atorvastatin, a classic drug for reducing cholesterol production by inhibiting 3-hydroxy-3-methyl-glutaryl-coenzyme A (HMG-CoA) reductase, could decrease serum HMGB1 and hs-CRP levels. Previous studies have demonstrated that statins reduce pro-inflammatory cytokines such as TNF-α, IL-6, CRP and there is crosstalk between HMGB1 and other pro-inflammatory cytokines, such as TNF-α, IL-6 and CRP (16–18). Recently, studies have reported that atorvastatin downregulates the expression of HMGB1 in brain ischemia and inhibits HMGB1-induced vascular endothelial activation (19,20). These results suggest that atorvastatin inhibits the expression of pro-inflammatory cytokines including HMGB1. A previous study showed that statins could provide cardioprotection for anti-atherosclerotic therapy (21). Thus, we speculated that the anti-atherosclerotic effect of atorvastatin may be attributed to the inhibition of HMGB1 in hyperlipidemia.

In conclusion, the present study showed that serum HMGB1 levels were increased in patients with hyperlipidemia, and that atorvastatin reduces serum HMGB1 levels which may be attributed to the decrease in serum lipids. Limitations were present in the present study. Overall, our study included only a small group of Chinese patients, therefore a future study with a large cohort is required. The precise mechanisms underlying our observation and their clinical relevance will require future elucidation.

## Figures and Tables

**Table I:** Characteristics of the hyperlipidemic and control patients.

	Control (n=32)	Hyperlipidemia (n=72)
Age (years)	48.2±9.5	49.4±10.3
Male (%)	46.8	51.4
Smokers (%)	31.3	34.7
Drinking (%)	21.9	20.8
Diabetes (%)	28.1	25.0
Hypertension (%)	34.4	36.1
Aspirin (%)	15.6	19.4
β-blocker (%)	6.3	6.9
Calcium blocker (%)	25.0	27.8
ACEI/ARB (%)	18.8	19.4
BMI (kg/m^2^)	23.9±3.3	25.0±4.1
TC (mmol/l)	4.34±0.89	6.37±0.94^a^
TG (mmol/l)	1.54±0.68	1.91±0.77
HDL (mmol/l)	1.06±0.31	0.98±0.35
LDL (mmol/l)	2.57±0.82	4.99±0.75^a^
hs-CRP (mg/l)	1.53±0.45	3.91±1.06^a^
HMGB1 (ng/ml)	2.11±0.95	5.42±1.56^a^

Data are presented as means ± SD. Smoking, ≥half pack/d within three months before this study; Drinking, ≥50g/d white wine within three months before this study; ACEI, angiotensin converting enzyme inhibitor; ARB, angiotensin receptor blocker; BMI, body mass index; TC, total cholesterol; TG, triglycerides; HDL-C, high-density lipoprotein cholesterol; LDL-C, low-density lipoprotein cholesterol; hs-CRP, high-sensitivity C-reactive protein; HMGB1, high mobility group box 1 protein.

a P<0.05, compared to the controls.

## Abbreviations:

TNF-α: tumor necrosis factor-α;
IL-6: interleukin-6;
CRP: C-reactive protein;
hs-CRP: high-sensitivity C-reactive protein;
HMGB1: high mobility group box 1 protein;
TC: total cholesterol;
TG: triglycerides;
HDL-C: high-density lipoprotein cholesterol;
LDL-C: low-density lipoprotein cholesterol
